# Intraoperative chlorhexidine-induced anaphylaxis suggesting an immunoglobulin-E-dependent mechanism indicated by basophil activation tests: two case reports

**DOI:** 10.1186/s40981-022-00581-w

**Published:** 2022-11-22

**Authors:** Masaki Orihara, Tomonori Takazawa, Tatsuo Horiuchi, Kazuhiro Nagumo, Noboru Maruyama, Akihiro Tomioka, Shigeru Saito

**Affiliations:** 1 Department of Anesthesiology, Gunma University Graduate School of Medicine, 3-39-22, Showa-Machi, Maebashi, 371-8511 Japan; 2 Intensive Care Unit, Gunma University Hospital, 3-39-15, Showa-Machi, Maebashi, 371-8511 Japan; 3 Department of Anesthesiology, Takasaki General Medical Center, 36, Takamatsu-Cho, Takasaki, 370-0829 Japan; 4 Department of Anesthesiology, JCHO Gunma Chuo Hospital, 1-7-13, Koun-Cho, Maebashi, 371-0025 Japan

**Keywords:** Anaphylaxis, Chlorhexidine, Skin test, Basophil activation test, Specific IgE

## Abstract

**Background:**

Although chlorhexidine allergy has been shown to be mediated by immunoglobulin (Ig) E, few reports investigated the mechanism of chlorhexidine-induced anaphylaxis using basophil activation tests (BATs).

**Case presentation:**

A 79-year-old man underwent cholecystectomy under general anesthesia. Anaphylaxis was diagnosed based on the clinical symptoms and high serum tryptase and histamine levels. Skin tests showed positive results only for chlorhexidine. Subsequently, BATs demonstrated that the causative agent was likely chlorhexidine. The inhibitory effect of wortmannin, an inhibitor of phosphoinositide 3-kinase, on basophil activation suggested an IgE-dependent mechanism underlying chlorhexidine-induced anaphylaxis.

An 89-year-old man underwent inguinal hernioplasty under general anesthesia. Anaphylaxis was diagnosed based on the clinical symptoms and high serum tryptase and histamine levels. Skin tests and BATs with wortmannin were performed, showing similar results to case 1.

**Conclusions:**

BATs suggested an IgE-dependent mechanism for chlorhexidine-induced anaphylaxis and might be useful for investigating the mechanisms underlying drug-induced anaphylaxis.

## Background

Chlorhexidine is an antiseptic with a broad spectrum of activity and persistent effects on the skin. Since the widespread use of chlorhexidine, many cases of anaphylaxis have been reported [1]. The risk of chlorhexidine allergy has increasingly been recognized in recent years [2]. Chlorhexidine was the third-most common cause of perioperative anaphylaxis after antibiotics and neuromuscular blocking agents [3]. The mechanisms underlying anaphylactic reactions to chlorhexidine have been investigated, and chlorhexidine allergy has been shown to be mediated by immunoglobulin (Ig) E [4]. To the best of our knowledge, this is the first report suggesting an IgE-dependent mechanism, as indicated by basophil activation tests (BATs).

## Case presentation

### Case 1

A 79-year-old man (weight, 73 kg; height, 161 cm) underwent cholecystectomy under general anesthesia. Anesthesia was induced with propofol, rocuronium, fentanyl, and inhalation of sevoflurane. Anesthesia was maintained with sevoflurane and remifentanil. He was given sulbactam and cefoperazone, and his skin was previously disinfected with chlorhexidine 1% (Hexak®; Yoshida Pharmaceutical Company, Tokyo, Japan). During surgery, frequent administrations of ephedrine and phenylephrine were required intraoperatively. Postoperatively, cutaneous wheals were observed, mainly over the abdomen. On suspicion of anaphylaxis, we administered 100 mg of hydrocortisone intravenously and 0.7 mg of adrenaline intramuscularly, stabilizing his general condition. He was transferred to the intensive care unit without extubation, and his trachea was extubated the next day. Serum tryptase levels measured at 30 min, 2 h, and 24 h after the reaction was identified were 9.6 μg/L, 10.0 μg/L, and 3.1 μg/L, respectively. Serum histamine levels measured at the same time points were 3.93 ng/L, 1.07 ng/L, and 1.27 ng/L, respectively. We diagnosed anaphylaxis based on the clinical symptoms and high serum tryptase and histamine levels.

**Fig. 1:**
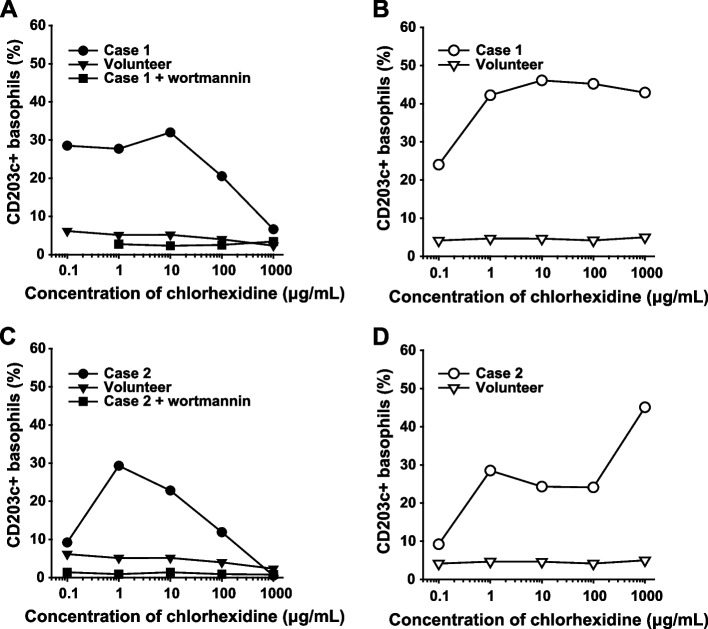
Results of basophil activation tests with CD203c. **A** Chlorhexidine (Hexak®) induced CD203c upregulation in case 1 (filled circles), but not in a volunteer (filled triangles). Wortmannin significantly suppressed chlorhexidine-induced CD203c upregulation (filled rectangles). **B** Chlorhexidine (Stericlon®)-induced CD203c upregulation in the patient (open circles), but not in a volunteer (open triangles). **C** Basophil activation tests in case 2 show similar results to **A**. **D** Basophil activation tests in case 2 show similar results to **B**

Eighty-three days later, skin tests were performed with all suspected agents, showing positive results only for chlorhexidine (Hexak®) (Table 1). BATs with CD203c and CD63 (Beckman Coulter, Brea, USA) were subsequently performed using serial dilutions of chlorhexidine (Hexak® and Stericlon®; KENEI Pharmaceutical Company, Osaka, Japan). Hexak® contains alcohol, but Stericlon® does not. The methods of the BATs are detailed elsewhere [5]. Briefly, the basophils were incubated at 37 °C for 15 min with antibodies and drugs. Anti-IgE antibody (Beckman Coulter) and formyl-methionine-leucyl-phenylamine (fMLP; Sigma-Aldrich, St. Louis, USA) were used as positive controls. The isotype of the anti-IgE antibody is IgG1, and the host species is a mouse. Unlike anti-IgE, fMLP activates basophils through an IgE-independent pathway [5, 6]. Wortmannin (Abcam, Cambridge, UK), an inhibitor of phosphoinositide 3-kinase (PI3-K), was used to investigate whether anaphylaxis was caused by an IgE-dependent or -independent mechanism [7]. We added one μM wortmannin at the same time as other drugs. Compared to CD203c + basophils from a healthy control, those in the patient were significantly elevated after adding Hexak® and Stericlon® (Fig. 1). Similar results were obtained for CD63 + basophils. These results showed that the causative agent was likely chlorhexidine. The rates of CD203c + basophils that responded to negative control, anti-IgE at 10 μg/mL, and fMLP at 1.3 μg/mL stimulation were 5.0%, 22.9%, and 17.3%, and those with pretreatment wortmannin were 3.2%, 2.5%, and 7.7%, respectively. Pretreatment with wortmannin inhibited basophil activation induced by anti-IgE and chlorhexidine. However, only a relatively small change was observed when fMLP was used as a basophil stimulator. The inhibitory effect of wortmannin on basophil activation suggested an IgE-dependent mechanism underlying chlorhexidine-induced anaphylaxis (Fig. 1). In addition, the level of specific IgE to chlorhexidine was measured at 6.63 UA/mL.

**Table 1:** Results of skin tests.

Drug	SPT				IDT			
	Concentration of stock solution (mg/mL)	Judgment	Wheal (mm)	Flare (mm)	Concentration of stock solution (mg/mL)	Judgment	Wheal (mm)	Flare (mm)
**Case 1**								
Saline	9	-			9	-		
Histamine	10	+	6	18	0.01	+	10	25
Propofol	10	-			1	-		
Rocuronium	10	-			0.05	-		
Remifentanil	0.05	-			0.005	-		
Fentanyl	0.05	-			0.005	-		
Sulbactam and cefoperazone	10	-			1	-		
Latex	NA	-			NA	-		
Chlorhexidine	5	-			0.002	+	6	20
**Case 2**								
Saline	9	-			9	-		
Histamine	10	+	5	8	0.01	+	9	12
Propofol	10	-			1	-		
Rocuronium	10	-			0.05	-		
Remifentanil	0.05	-			0.005	-		
Fentanyl	0.05	-			0.005	-		
Sulbactam and ampicillin	15	-			1.5	-		
Acetaminophen	10	-			1	-		
Hydroxyethyl starch	60	-			6	-		
Chlorhexidine	5	+	10	12	0.002	+	6	10

SPT reactions were deemed positive for wheal diameters ≥ 3 mm larger than that of the negative control or equal to at least half that in the positive control after 20 min. IDT reactions were deemed positive when the wheal diameter was equal to at least twice that of the post-injection wheal after 20 min [8]. *SPT*, skin prick test; *IDT*, intradermal test; *NA*, not applicable

### Case 2

An 89-year-old man (weight, 47 kg; height, 160 cm) underwent inguinal hernioplasty under general anesthesia. Anesthesia was induced with propofol, rocuronium, fentanyl, and inhalation of desflurane. Anesthesia was maintained with desflurane and remifentanil. He was given sulbactam and ampicillin, and his skin had been previously disinfected with chlorhexidine 0.5% (Hexak®). Blood pressure fell to approximately 50 mmHg in the middle of the operation. Suspecting anaphylaxis, a total of 0.6 mg of adrenaline, chlorpheniramine 5 mg, and famotidine 20 mg were administered, resulting in stabilization of his general condition. Generalized erythema was observed when the surgery was concluded. Since he did not show any respiratory symptoms at the end of anesthesia, he was transferred to the high care unit after extubation. Serum tryptase levels measured at 30 min, 2 h, and 24 h after the reaction were 11.0 μg/L, 18.9 μg/L, and 4.9 μg/L, respectively. Serum histamine levels measured at the same time points were 67.1 ng/L, 2.94 ng/L, and 0.81 ng/L, respectively. Anaphylaxis was diagnosed based on the clinical symptoms and high serum tryptase and histamine levels.

Thirty-four days later, skin tests and BATs with wortmannin were performed, showing similar results to case 1 (Table 1, Fig. 1). Rates of CD203c + basophils that responded to negative control, anti-IgE 10 μg/mL, and fMLP 1.3 μg/mL stimulation were 5.0%, 68.9%, and 59.1%, and those with wortmannin were 3.6%, 3.8%, and 58.6%, respectively. In addition, the level of specific IgE to chlorhexidine was 1.45 UA/mL.

## Discussion

We report two cases of chlorhexidine-induced anaphylaxis diagnosed by skin tests, BATs, and specific IgE quantification. BATs suggested an IgE-dependent mechanism for chlorhexidine-induced anaphylaxis.

Correct diagnosis is crucial in perioperative anaphylaxis, and skin tests remain the gold standard for detecting culprit drugs. However, skin tests carry a risk of inducing a recurrence of anaphylaxis, and the positive predictive value of skin tests is not 100%. Hence, there seems to be room for other in vitro tests to diagnose anaphylaxis [9]. The following in vitro tests are available to assess the diagnosis of chlorhexidine allergy: detection of specific IgE, histamine release test, and BATs. Although many patients have undergone quantification of specific IgE, few patients with chlorhexidine-induced anaphylaxis have undergone BATs [1]. We performed BATs in addition to specific IgE in our cases, with positive results in both. The positive reaction on BATs by chlorhexidine preparations with or without alcohol suggested that chlorhexidine itself is an allergen.

In recent years, BATs with wortmannin have become available to investigate the involvement of IgE. As PI3-K is an essential enzyme in the IgE-mediated pathway of basophil activation, suppression of basophil activation by wortmannin suggests IgE involvement [7, 10]. In the current cases, wortmannin almost completely suppressed basophil activation by chlorhexidine, suggesting an IgE-dependent mechanism of chlorhexidine-induced basophil activation.

## Conclusions

BATs suggested an IgE-dependent mechanism for chlorhexidine-induced anaphylaxis. BATs might be useful for investigating the mechanisms underlying drug-induced anaphylaxis.

## Data Availability

Data relevant to this case report are not available for public access because of patient privacy concerns, but are available from the corresponding author on reasonable request.

## Abbreviations

BATs: Basophil activation tests
IgE: Immunoglobulin-E
PI3-K: Phosphoinositide 3-kinase
fMLP: Formyl-methionine-leucyl-phenylamine
